# Incidence and Long-Term Outcomes of Biliary Tract Cancers in Olmsted County, Minnesota from 1976 to 2018

**DOI:** 10.3390/cancers16152720

**Published:** 2024-07-31

**Authors:** Eugene C. Nwankwo, Amerti Guta, Scarlett S. Cao, Ju Dong Yang, Abubaker Abdalla, Wesam Taha, Joseph J. Larson, Jun Yin, Gregory J. Gores, Sean P. Cleary, Lewis R. Roberts

**Affiliations:** 1 Division of Gastroenterology and Hepatology, Mayo Clinic, Rochester, MN 55905, USA; 2 Center for Clinical and Translational Science, Mayo Clinic, Rochester, MN 55905, USA; 3 Division of Gastroenterology, Hepatology and Nutrition, Vanderbilt University Medical Center, Nashville, TN 37232, USA; 4 Department of Internal Medicine, Mayo Clinic School of Graduate Medical Education, Rochester, MN 55902, USA; 5 Division of Gastroenterology and Hepatology, Cedars-Sinai Medical Center, Los Angeles, CA 90048, USA; 6 Department of Medicine, Emory University School of Medicine, Atlanta, GA 30322, USA; 7 Division of Hospital Internal Medicine, Mayo Clinic, Rochester, MN 55905, USA; 8 Department of Internal Medicine, New York Presbyterian, Flushing, NY 11355, USA; 9 Division of Clinical Trials and Biostatistics, Mayo Clinic, Rochester, MN 55905, USA; 10 Division of General Surgery, Department of Surgery, University of Toronto, Toronto, ON M5S 1A1, Canada

**Keywords:** intrahepatic cholangiocarcinoma, extrahepatic cholangiocarcinoma, epidemiology, incidence, survival

## Abstract

**Simple Summary:**

Biliary tract cancers, which include cholangiocarcinoma, gallbladder, and ampulla of Vater cancers, are a significant health concern due to their increasing incidence and poor survival rates. Using data from the Rochester Epidemiology Project, this study tracks changes in incidence and survival rates among residents aged 20 and over in Olmsted County, Minnesota from 1976 to 2018. The findings illustrate a rise in overall biliary tract cancers from 6.53 cases to 10.25 cases per 100,000 person-years between 1976 and 2018 (*p* = 0.004). The incidence of intrahepatic cholangiocarcinoma has significantly increased from 0.25 to 3.93 per 100,000 person-years over the four decades, with a more pronounced increase observed in males, while gallbladder cancer incidence has decreased among women. Despite improvements in median survival times from 4.2 months to 10.8 months over the four decades (*p* = 0.019), the overall prognosis remains very poor. This study highlights the evolving epidemiology of these cancers and the critical need for enhanced diagnostic and treatment strategies to improve patient outcomes.

**Abstract:**

Biliary tract cancers, including cholangiocarcinoma, gallbladder, and ampulla of Vater cancers, rank second among hepatobiliary cancers, known for their poor prognoses. The United States has witnessed a notable increase in intrahepatic cholangiocarcinoma incidence. This study examines the incidence and survival outcomes of biliary tract cancers in Olmsted County, Minnesota from 1976 to 2018. Using data from the Rochester Epidemiology Project (REP), residents aged 20 and above were analyzed across four eras. Incidence rates were calculated and adjusted for age and sex, and temporal trends were assessed using Poisson regression. Intrahepatic cholangiocarcinoma exhibited a significant escalation in incidence rates over time, while gallbladder cancers showed a decline among women. Median survival times for biliary tract cancers notably improved. These findings confirm the rising incidence of intrahepatic cholangiocarcinoma and suggest improving survival rates. Nevertheless, the overall prognosis for biliary tract cancers remains very poor, emphasizing the continual need for enhanced management strategies and further research.

## 1. Introduction

Biliary tract cancers—malignancies arising from the biliary system—are a heterogeneous group of malignancies including intrahepatic, perihilar, and distal cholangiocarcinoma, gallbladder cancer, and ampulla of Vater cancers [1,2,3]. These cancers account for approximately 3% of all gastrointestinal neoplasms and are the sixth most common cancer of the digestive tract globally [4,5]. These cancers are rare and, in part due to their late presentations at advanced stages of the disease, their overall survival rates remain very poor [3,6].

The epidemiology of these cancers has been changing over time. Most prominently for intrahepatic cholangiocarcinoma, multiple studies using SEER and WHO databases have seen an increase in incidence and mortality rates over the past several decades [7,8,9,10]. In contrast, gallbladder cancer incidence has declined in recent decades, with some improvement in survival rates in these patients [4,11,12,13,14]. Other types of biliary tract cancers, like extrahepatic cholangiocarcinoma, have also shown some decline in mortality rates [13,15,16].

Along with changes in the epidemiology of biliary tract cancers in recent decades, there have also been significant changes in treatment. Non-surgical options for unresectable or locally advanced biliary tract cancers have now advanced to include local and locoregional therapies such as radiofrequency and microwave ablation, transarterial chemoembolization and radioembolization, external beam radiation therapy with stereotactic body radiation therapy or proton beam therapy, and brachytherapy [17,18,19,20]. Additionally, changes in systemic chemotherapy to include pembrolizumab, gemcitabine, and cisplatin for advanced or metastatic biliary tract cancers have shown survival advantages in a phase 3 clinical trial [21].

Biliary tract cancers are influenced by various risk and prognostic factors [22]. Key risk factors include a history of inflammatory bowel disease with primary sclerosing cholangitis, cirrhosis, and chronic viral hepatitis, all of which cause chronic inflammation and bile duct damage [23,24,25,26]. Additional risk factors include the presence of gallstones and a porcelain gallbladder, which are linked to chronic biliary inflammation [27]. Lifestyle factors such as alcohol use and smoking, along with a family history of liver or bile duct cancer, also increase the risk, with smoking and alcohol exacerbating liver damage and cirrhosis, thereby worsening the prognosis [28]. In this study, we examine the trends in biliary tract cancer incidence rates and survival in Olmsted County, Minnesota between the years of 1976 and 2018, expanding on previous work to further delineate changes in biliary tract cancer epidemiology over time [29].

## 2. Materials and Methods

### 2.1. Database

The Rochester Epidemiology Project (REP) database was used to access the medical records of patients in Olmsted County, Minnesota. Most of the healthcare in Olmsted County is delivered through 3 facilities—the Mayo Clinic, Olmsted Medical Center, and Rochester Family Medicine Clinic—all of which use a unit or dossier medical record system that assembles all patient data in one place. The REP is a medical linkage system established in 1966 that shares the medical records of these facilities for research purposes under its umbrella [30].

This database maintains indexes of demographic data, medical diagnosis, and surgical procedure codes used to identify study patients. Data can be abstracted directly from each patient’s medical chart—encompassing details of outpatient visits, hospitalizations, and emergency room visits, in addition to laboratory data and pathology reports [30].

The major advantages of the REP are that the system captures virtually the entire population of Olmsted County, which is relatively isolated and has a considerable longitudinal span, with records that are continually updated. Follow-up with REP providers is also high, with 93% of Olmsted County residents having been seen by a provider within 3 years [30].

This study was approved by the institutional review boards responsible for the REP—the Mayo Clinic and Olmsted Medical Center IRBs.

### 2.2. Study Setting

Olmsted County is located in southeastern Minnesota and had a population of 162,847 as of the 2022 US Census. The majority of patients (76.6%) identified as non-Hispanic whites. In 2022, the median age of inhabitants was 37.9, compared to 38.9 nationally. Approximately 36% of the population was older than 50 years of age, and 50.91% were female. These results were similar to national census data indicating that the proportion of the United States population older than 50 years of age is 36%, with 50.4% being female. This is slightly higher than the national average, where about 34% of the population is older than 50 years of age. In regards to socioeconomic factors, 30% of Olmsted County residents had attained a minimum of an undergraduate degree or equivalent, compared to a national average of 21% [31].

### 2.3. Study Design and Study Population

A total of 205 Olmsted County residents aged 20 and over with a new diagnosis of biliary tract cancer from 1 January 1976 to 31 December 2018 were retrospectively identified through the REP. Candidates were identified by HICDA, ICD-9, and ICD-10 codes. The list of codes used are available in Supplemental Table S1. To exclude those who may have moved to the county to pursue medical care, only candidates who had lived in Olmsted County for 1 year prior to diagnosis were included.

Biliary tract cancers were classified into 5 subtypes based on anatomic location: intrahepatic cholangiocarcinoma, perihilar cholangiocarcinoma, distal cholangiocarcinoma, ampulla of Vater cancer, and gallbladder cancer. Intrahepatic cholangiocarcinoma was defined as a mass-forming tumor in the liver parenchyma arising from the small intrahepatic bile ductules beyond the secondary biliary radicals. Perihilar cholangiocarcinoma was defined as a tumor involving the common hepatic duct, bifurcation of the hepatic duct, and large intrahepatic ducts up the secondary biliary radicals.

Diagnosis of biliary tract cancer was made by histology, cytology, or characteristic radiographic features on CT, MRI, or ERCP. Characteristic radiographic features included tumors arising from the biliary tract on cross-sectional imaging, without evidence of primary origin in other areas of the GI tract or pancreas, with malignant progression over time.

### 2.4. Clinical Information

For those patients who qualified for this study, medical records were reviewed for data including age, gender, race, and associated risk factors including liver cirrhosis, viral hepatitis B and C, primary sclerosing cholangitis, alcohol use, smoking status, family history of biliary tract cancer, history of gallstones, and porcelain gallbladder diagnosis.

Tumor staging, primary treatment modality, survival status, and cause of death were also abstracted from the patient’s medical records. For tumor staging, surgical TNM staging was used primarily if available; otherwise, clinical TNM staging based on cross-sectional imaging at the time of diagnosis was used.

The primary treatment modalities for biliary tract cancers were classified into 3 categories: surgical resection and liver transplantation; systemic chemotherapy or locoregional therapy without surgery; and comfort care only. Vital status was assessed as of 1 September 2019. Cause of death was classified as biliary tract cancer-related—defined as death from a complication of the biliary tract cancer as a main or contributing factor—or non-cancer related.

### 2.5. Statistics

Patient demographics and clinical characteristics were summarized by the era and subtype of biliary tract cancer. Age at diagnosis was summarized by median and range, and the Kruskal–Wallis test was used for assessing differences between eras and subtypes. Diagnosis methodology, risk factors, stage, and treatment modality were summarized with counts and percentages. The Chi-square T test was used to test for associations between these categorical variables by era and subtype. Missing data were tabulated for each demographic, risk factor, and clinical characteristic. Patients with missing data were excluded from any analyses involving those characteristics.

In biliary tract cancer incidence rate calculations, the entire population of Olmsted County aged 20 and over was considered at risk. Age-specific person-years were estimated by decennial census data for Olmsted County with linear interpolation between census years. Incidence rates and 95% confidence intervals for incidence rates were calculated with the assumption that incident cases follow a Poisson distribution and summarized per 100,000 person-years. The trend for incidence rates was tested by fitting a Poisson regression model where cancer counts were the outcome, and age, sex, and time period were independent predictors. Each model was adjusted for the population size of each period.

As this study builds off of the previous work of this study team, the previously reported era of 2001–2008 was expanded to 2001–2010 to complete the decade. The data were collected in the same database using the same methods, and the analysis methods were performed in an identical fashion to our previous paper.

Overall survival was defined as the time from cancer diagnosis to last-known follow-up or death. Survival was analyzed using the Kaplan–Meier method, and a log-rank test was used for comparisons between eras and subtypes.

R (v. 3.6.2, Vienna, Austria) and SAS (v.9.4, SAS Institute, Cary, NC, USA) were used for statistical analysis. An alpha of 0.05 was used for all tests.

## 3. Results

### 3.1. Patient Characteristics

Through the REP database, patients were identified using the Hospital International Classification of Disease Adaptation (HICDA) and the International Classification of Disease (ICD) codes. The ICD-9 and ICD-10 codes for biliary tract cancers were implemented for diagnoses made between 1976 and 2018. For those identified, patient records were reviewed and, after excluding those without biliary tract cancer and not meeting residency criteria, 205 patients with biliary tract cancer were qualified for this study.

Patient characteristics are listed in Table 1. The median age of diagnosis was 70.3 years for overall biliary tract cancers. The cohort was predominantly white (96.6%), with a small male majority (51.2%). Comparing characteristics by subtypes, notably for intrahepatic cholangiocarcinoma, the median age of diagnosis was younger at 66.5 years, and most patients were male (63.8%). This contrasts with gallbladder cancers, where most patients were female (62.3%).

Supplemental Table S2 shows further details, including patient characteristics, diagnostic, and treatment information categorized into four decennial periods from 1976 to 2018. The median age at diagnosis for the biliary tract cancer subtypes remained relatively stable with slight fluctuation across the different time periods. For intrahepatic CCA, the median age ranged from 59.6 years (1991–2000) to 65.6 years (2011–2018). Perihilar CCA exhibited a gradual increase in median age at diagnosis, from 66.3 (1976–1990) to 81.3 (2011–2018). The median age of diagnosis for distal CCA ranged from 63.5 years (1991–2000) to 78.2 years (2001–2010), with an overall median age of diagnosis at 70.2, and immediate fluctuations between the eras. Similarly, the overall median age of diagnosis for the ampulla of Vater cancer is 70.4 years old and stayed stable through the years from 74.5 (1976–2000) to 73.6 (2011–2018). Gallbladder cancer showed a relatively high median age at diagnosis over the years compared to the other subtypes of biliary tract cancer, with an overall median age at diagnosis of 72.8 years.

For the associated risk factors by subtype (Table 1), a significant portion (23.4%) of patients with intrahepatic cholangiocarcinoma had cirrhosis. In perihilar cholangiocarcinoma, a comparatively higher proportion of patients (12.8%) had primary sclerosing cholangitis. For gallbladder cancer, most patients had a history of gallstones (73.8%), and a small percentage (4.9%) had porcelain gallbladder. Overall, most patients (99.0%) did not have any family history of biliary tract cancer. No statistically significant difference was noted in alcohol use, hepatitis B or C, or smoking between subtypes.

### 3.2. Incidence Rates of Biliary Tract Cancers

For overall biliary tract cancers, the age- and sex-adjusted incidence rates increased from 6.53 cases to 10.25 cases per 100,000 person-years between 1976 to 1990 and 2011 to 2018 (*p* = 0.004) (Table 2). This increase appears to be more prominently seen in males (*p* = 0.001).

By subtype, intrahepatic cholangiocarcinoma demonstrates a progressive increase in the age- and sex-adjusted incidence rates across eras, from 0.25 cases to 3.93 cases per 100,000 person-years from 1976 to 1990 to 2011 to 2018 (*p* < 0.001). While this increase has been observed in both sexes, this appears to have been more prominently seen in males (*p* < 0.001).

Notably, gallbladder cancers did not have a statistically significant change overall in incidence across eras (*p* = 0.266); however, a declining incidence rate of these cancers was noted in women—declining from 3.88 to 1.52 cases per 100,000 person-years between 1976 to 1990 and 2011 to 2018 (*p* = 0.015). Additionally, perihilar cholangiocarcinoma (*p* = 0.651), distal cholangiocarcinoma (*p* = 0.594), and ampullary cancers (*p* = 0.176) did not show a statistically significant change in incidence rates across eras (Supplemental Figure S1).

### 3.3. Initial Treatment and Outcomes of Biliary Tract Cancers

Regarding the stage of overall biliary tract cancers, the majority of patients (62.9%) were diagnosed at late stages III/IV (Table 3). Gallbladder carcinomas (90.2%), perihilar cholangiocarcinoma (62.2%), and intrahepatic cholangiocarcinoma (55.3%) were more often diagnosed at later stages III/IV. Not unexpectedly, distal cholangiocarcinoma and ampullary cancers were more often diagnosed at earlier stages I/II, with results of 70.8% and 56.0%, respectively. Notably, in examining whether the stage of diagnosis differed across the four eras, there was no statistically significant difference observed (*p* = 0.623), as most patients were still diagnosed at late stages III/IV (Table 4).

Regarding treatment, ampullary cancers (76.9%), gallbladder cancers (47.5%), and distal cholangiocarcinoma (43.5%) had higher proportions of patients undergoing surgical management (Table 3). Over the past decade, there has been a shift to more use of chemotherapy or localized therapy, which were used in around 47.9% of cases in the most recent period (Table 4).

**Figure 1:**
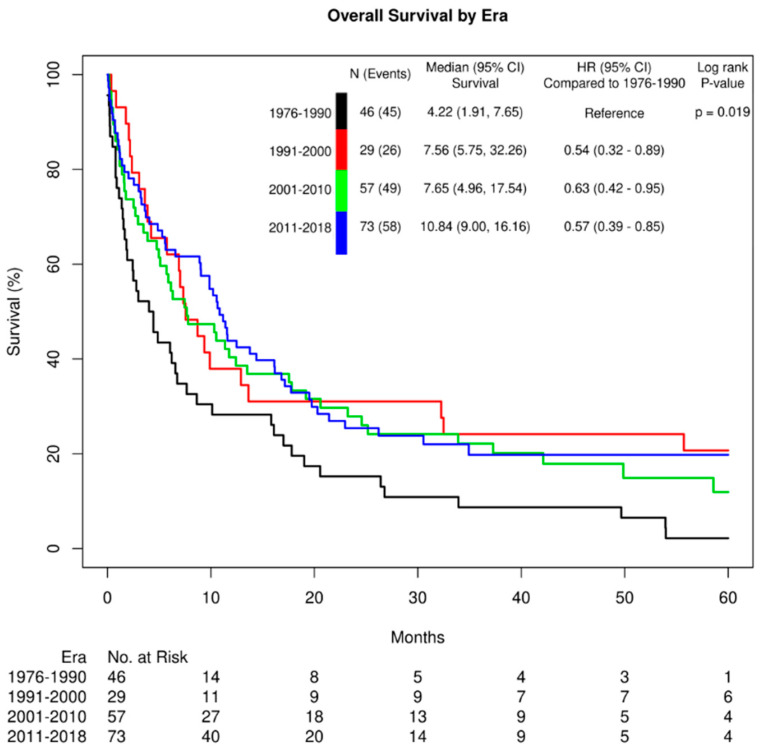
Overall biliary tract cancer survival by era.

Overall survival continues to be poor amongst all subtypes of biliary tract cancers, with a small survival advantage found with ampullary cancers (Table 3). In comparing all biliary tract cancers across eras, there was a statistically significant increase in median survival time from 4.2 months to 10.8 months between 1976 to 1990 and 2011 to 2018 (*p* = 0.019) (Figure 1). However, there was no significant change in median survival noted among the individual subtypes between 1976 to 1990 and 2011 to 2018 (Supplemental Figure S2; Supplemental Table S3).

## 4. Discussion

This study outlines the epidemiology of biliary tract cancers in the population of Olmsted County, Minnesota over the span of four decades, specifically highlighting the increase in intrahepatic cholangiocarcinoma in the population, as well as the improved median survival rate of patients over time.

Recent studies have noted an overall trend towards increased mortality rates for biliary tract cancers, particularly due to intrahepatic cancers [5,32]. Similarly, our study noted that intrahepatic cholangiocarcinoma incidence increased from 0.25 to 3.93 cases per 100,000 persons between the 1976 to 1990 and 2011 to 2018 time periods. These data corroborate similar trends in intrahepatic cholangiocarcinoma incidence rates from multiple other studies [7,8,9,10]. In work conducted using the Surveillance, Epidemiology, and End Results (SEER) database between 1973 and 1997, the incidence rate of intrahepatic cholangiocarcinoma was noted to increase from 0.13 to 0.67 cases per 100,000 persons, with an estimated annual percent change (EAPC) of 9.11% [33]. Later work using the SEER data between 1973 and 2012 also noted a rise in intrahepatic cholangiocarcinoma from 0.44 to 1.18 cases per 100,000 [8]. This has been similarly supported by work using the North American Association of Central Cancer Registries database between 1999 and 2013 when intrahepatic cholangiocarcinoma incidence increased across gender and racial groups, with an EAPC of 3.2% [12].

In prior studies, the observed rise in intrahepatic cholangiocarcinoma incidence was thought to be partially confounded by the ICD code classification of Klatskin tumors as intrahepatic cholangiocarcinoma; this has been disputed as previous work has indicated that even with the exclusion of Klatskin tumors, intrahepatic cholangiocarcinoma incidence rates are experiencing a true increase [34]. Our results likewise suggest that there is a true increase in the incidence of intrahepatic cholangiocarcinoma in the Olmsted County population. Our ability to perform a detailed independent review of individual medical records allows us to correct any errors in code classification. Furthermore, the proportions of patients diagnosed with early-stage disease have remained stable across eras.

As a potential explanation for the increase in the incidence of intrahepatic cholangiocarcinoma, our data have demonstrated that in patients with intrahepatic cholangiocarcinoma, a large proportion had underlying cirrhosis compared to other subtypes of biliary tract cancer. Cirrhosis has been noted to be a significant risk factor linked to cholangiocarcinoma [35,36,37], with one study noting a 10-fold increased risk of patients with cirrhosis developing cholangiocarcinoma [35,36]. The prevalence of cirrhosis has also been increasing in the United States over the past two decades [36,38]. Notably, the patients in our study did not have significantly increased alcohol use or hepatitis B or C histories, suggesting a potential role for other etiologies, including metabolic dysfunction associated steatotic liver disease.

In this study, we noted a trend of declining gallbladder cancer incidence rates over time in women, with more recent stabilization from 3.88 to 1.52 cases per 100,000 persons. The overall incidence rate has not changed significantly. Other studies have also noted similar trends—between 1974 and 2009, declining gallbladder cancer rates across both genders and most ethnicities were found using SEER data [14]. Similarly, an examination of gallbladder incidence rates between 1999 and 2011 using the U.S. Cancer Statistics dataset noted a declining incidence rate in women, with an EAPC of −0.5%, while the rates were stable among men [39]. This decline in gallbladder cancer incidence has been inversely linked to an increased rate of cholecystectomies in the general population, which may mitigate the development of high-risk gallbladder disease [40].

Interestingly, the median survival for all biliary tract cancers in this study, while still poor, has significantly improved over time. The latest era from 2011 to 2018 has a higher median survival time of 10.8 months, compared with 4.2 months in the earliest era from 1976 to 1990. This is not accompanied by any significant difference in disease stage, with most cases diagnosed at the late stages III or IV throughout all eras, suggesting against lead-time bias. The majority of patients were not initially treated surgically, and the increase in non-surgical management for advanced biliary tract cancers, particularly increased use of systemic chemotherapy and locoregional therapies over time, may be partially responsible for the improvement in survival. This improvement in survival has not been widely reported previously, but with the rise in intrahepatic cholangiocarcinoma incidence, other studies have seen improvement in intrahepatic cholangiocarcinoma survival more recently linked to nonoperative therapy [41].

The major strength of this study derives from the comprehensive and detailed clinical data provided by the REP. The medical records encompass essentially the entire Olmsted County population, and in this study, the individual records were independently reviewed to accurately classify biliary tract cancer subtypes. This is a substantial benefit compared to other studies that have relied on diagnostic codes, which have been subject to change.

One limitation of this study is the generalizability of the population of Olmsted County to the general population of the U.S. The Olmsted County population is predominantly white and has access to highly specialized healthcare through the Mayo Clinic, which may have had some influence on the detection and subtype classification of these cancers. Also, despite the increasing incidence of biliary tract cancers, given the low county population of 140,000, the relatively small number of cases limits the level of detail that can be achieved in the analysis of the biliary tract cancer subtypes. Furthermore, our study is retrospective in nature. Future studies focused on a prospective randomized approach would provide additional details on the impact of treatment and diagnostic modalities on the incidence and survival of various BTC subtypes. These limitations, however, must be weighed against the comprehensive advantages noted above. Overall, this study highlights the increasing incidence of biliary tract cancers, primarily due to the remarkably increasing trend in the incidence of intrahepatic cholangiocarcinoma, as well as a substantial improvement in survival for patients with biliary tract cancers over the 42-year span of this study. While improved, the overall prognosis for these cancers remains poor.

Recent advancements in treatment options such as targeted therapies have significantly improved patient outcomes. For instance, a substantial percentage of intrahepatic cholangiocarcinomas have been shown to bear mutations in the isocitrate dehydrogenase 1 and 2 genes or express oncogenic fusion proteins between the fibroblast growth factor receptor 2 (FGFR2) gene and several partner proteins [42,43]. These observations have led to the development of novel targeted treatments, such as ivosidenib [44] and pemigatinib [45], against the mutant isocitrate dehydrogenase genes and the FGFR2 fusion proteins, respectively [46]. Despite these advances in systemic and targeted therapy [47], the effective diagnosis of biliary tract cancers and cholangiocarcinoma (CCA) remains complex due to several factors. One major challenge is the lack of routine screening for this relatively rare cancer. While some patients may present with no symptoms, others experience non-specific signs and symptoms such as persistent abnormalities in liver function tests, unexplained weight loss, and upper abdominal discomfort. The onset of these symptoms can be delayed by several months or even years. Furthermore, a recent survey noted that a significant number of patients are initially misdiagnosed, often with other GI cancers, leading to delayed diagnosis and potentially impacting treatment options [48].

Further investigation is still needed, as there is still limited understanding of predisposing environmental and genetic risk factors to developing biliary tract cancers. Genome-wide association studies (GWAS) have been able to identify genetic variants for other cancers and are still needed in the study of biliary tract cancers. Through these studies, we may be able to better identify individuals at higher risk of these cancers, allowing earlier-stage diagnosis and the earlier use of therapeutic interventions [49]. These studies may also reveal key molecular pathways driving the pathogenesis of biliary tract cancers, allowing the development of more effective targeted therapies against these lethal cancers.

## 5. Conclusions

Our study elucidates the evolving epidemiology of biliary tract cancers within the Olmsted County, Minnesota population across four decades. Notably, there is a substantial rise in the incidence of intrahepatic cholangiocarcinoma, consistent with findings from broader databases such as SEER and NAACCR. While previously thought to be confounded by coding discrepancies, our detailed review confirms a genuine increase in intrahepatic cholangiocarcinoma incidence. This rise aligns with the growing prevalence of cirrhosis, suggesting potential etiological links. Conversely, gallbladder cancer rates, particularly in women, demonstrate a declining trend, potentially attributed to increased cholecystectomies. Despite the grim prognosis historically associated with biliary tract cancers, our study reveals a significant improvement in median survival, possibly attributable to advancements in non-surgical management strategies. Despite limitations in generalizability, our comprehensive approach underscores the importance of detailed clinical data in understanding cancer epidemiology. Moving forward, further investigations, including GWAS, are imperative to unravel the complex genetic and environmental factors underlying biliary tract cancers, paving the way for more effective therapeutic interventions.

## Figures and Tables

**Table 1:** Demographics, method of diagnosis, risk factors for study population. CCA, cholangiocarcinoma; BTC, biliary tract cancer.

	Intrahepatic CCA (N = 47)	Perihilar CCA (N = 47)	Distal CCA (N = 24)	Ampulla of Vater CA (N = 26)	Gallbladder CA (N = 61)	Total BTC (N = 205)	*p*-Value
**Demographics**							
Median Age (range)	66.5 (41.2, 96.5)	70.9 (37.0, 96.3)	70.2 (41.7, 94.8)	70.4 (38.5, 89.7)	72.8 (44.6, 89.8)	70.3 (37.0, 96.5)	0.193
Male, N (%)	30 (63.8%)	24 (51.1%)	13 (54.2%)	15 (57.7%)	23 (37.7%)	105 (51.2%)	0.093
White, N (%)	43 (91.5%)	47 (100.0%)	23 (95.8%)	26 (100.0%)	59 (96.7%)	198 (96.6%)	0.176
**Diagnosis**							0.111
Histology, N (%)	37 (78.7%)	28 (59.6%)	18 (75.0%)	25 (96.2%)	45 (73.8%)	153 (74.6%)	
Cytology, N (%)	8 (17.0%)	13 (27.7%)	4 (16.7%)	1 (3.8%)	11 (18.0%)	37 (18.0%)	
Radiology, N (%)	2 (4.3%)	6 (12.8%)	2 (8.3%)	0 (0.0%)	5 (8.2%)	15 (7.3%)	
**Risk Factors**							
Porcelain Gallbladder, N (%)	0 (0.0%)	0 (0.0%)	0 (0.0%)	0 (0.0%)	3 (4.9%)	3 (1.5%)	0.126
Gallstones, N (%)	23 (48.9%)	22 (46.8%)	10 (41.7%)	12 (46.2%)	45 (73.8%)	112 (54.6%)	0.011
Primary Sclerosing Cholangitis, N (%)	2 (4.3%)	6 (12.8%)	0 (0.0%)	0 (0.0%)	1 (1.6%)	9 (4.4%)	0.024
Cirrhosis, N (%)	11 (23.4%)	5 (10.6%)	0 (0.0%)	0 (0.0%)	0 (0.0%)	16 (7.8%)	<0.001
Alcohol Use, N (%)	6 (12.8%)	4 (8.5%)	3 (12.5%)	2 (7.7%)	5 (8.2%)	20 (9.8%)	0.900
Smoking, N (%) *	26/46 (56.5%)	31/47 (66.0%)	16/23 (69.6%)	15/26 (57.7%)	29/60 (48.3%)	117/200 (57.9%)	0.306
Family History, N (%) *	0/45 (0.0%)	0/46 (0.0%)	0/24 (0.0%)	0/26 (0.0%)	2/60 (3.3%)	2/200 (1.0%)	0.314
HBV, N (%)	2 (4.3%)	0 (0.0%)	0 (0.0%)	0 (0.0%)	0 (0.0%)	2 (1.0%)	0.147
HCV, N (%)	1 (2.1%)	0 (0.0%)	0 (0.0%)	0 (0.0%)	0 (0.0%)	1 (0.5%)	0.497

* Denominators shown due to missing data.

**Table 2:** Incidence rate of biliary tract cancers—cases per 100,000 person-years—by era. CCA, cholangiocarcinoma; BTC, biliary tract cancer.

	1976–1990 (N = 46)	1991–2000 (N = 29)	2001–2010 (N = 57)	2011–2018 (N = 73)	*p*-Value
**Intrahepatic CCA**					
**Age/Sex Adjusted**	0.25 (0.00, 0.60)	0.59 (0.01, 1.17)	1.49 (0.68, 2.31)	3.93 (2.45, 5.42)	<0.001
**Male**	0.29 (0.00, 0.85)	0.71 (0.00, 1.72)	2.70 (1.00, 4.39)	5.29 (2.71, 7.87)	<0.001
**Female**	0.23 (0.00, 0.68)	0.59 (0.00, 1.41)	0.55 (0.00, 1.19)	3.07 (1.24, 4.90)	0.002
**Perihilar CCA**					
**Age/Sex Adjusted**	1.68 (0.72, 2.63)	1.45 (0.50, 2.40)	1.26 (0.51, 2.02)	2.13 (1.02, 3.24)	0.651
**Male**	1.75 (0.34, 3.15)	2.22 (0.41, 4.04)	1.04 (0.01, 2.07)	2.48 (0.73, 4.23)	0.979
**Female**	1.42 (0.27, 2.57)	0.92 (0.00, 1.96)	1.46 (0.37, 2.55)	1.98 (0.49, 3.47)	0.509
**Distal CCA**					
**Age/Sex Adjusted**	0.96 (0.19, 1.74)	0.47 (0.00, 1.00)	0.96 (0.28, 1.64)	0.99 (0.24, 1.74)	0.594
**Male**	1.78 (0.00, 3.58)	0.00 (0.00, 0.00)	1.79 (0.35, 3.22)	1.00 (0.00, 2.14)	0.991
**Female**	0.55 (0.00, 1.31)	0.90 (0.00, 1.91)	0.34 (0.00, 0.85)	1.05 (0.01, 2.08)	0.486
**Ampulla of Vater CA**					
**Age/Sex Adjusted**	0.57 (0.01, 1.12)	0.51 (0.00, 1.09)	1.43 (0.61, 2.24)	1.12 (0.28, 1.96)	0.176
**Male**	0.87 (0.00, 2.10)	0.31 (0.00, 0.93)	2.43 (0.82, 4.03)	1.00 (0.00, 2.14)	0.344
**Female**	0.46 (0.00, 1.10)	0.62 (0.00, 1.49)	0.63 (0.00, 1.37)	1.28 (0.01, 2.54)	0.342
**Gallbladder CA**					
**Age/Sex Adjusted**	3.07 (1.78, 4.37)	1.59 (0.60, 2.58)	1.53 (0.69, 2.37)	2.08 (1.05, 3.11)	0.266
**Male**	2.02 (0.19, 3.85)	0.74 (0.00, 1.77)	1.67 (0.31, 3.03)	3.26 (1.23, 5.29)	0.195
**Female**	3.88 (2.02, 5.74)	2.17 (0.65, 3.70)	1.56 (0.40, 2.73)	1.52 (0.29, 2.75)	0.015
**Overall BTC**					
**Age/Sex Adjusted**	6.53 (4.63, 8.44)	4.61 (2.92, 6.30)	6.67 (4.92, 8.41)	10.25 (7.85, 12.65)	0.004
**Male**	6.70 (3.48, 9.92)	3.98 (1.58, 6.38)	9.62 (6.40, 12.84)	13.03 (8.97, 17.09)	0.001
**Female**	6.54 (4.09, 8.98)	5.20 (2.78, 7.62)	4.54 (2.60, 6.48)	8.89 (5.77, 12.02)	0.455

**Table 3:** Stage, treatment, and survival by biliary tract cancer subtypes. CCA, cholangiocarcinoma; BTC, biliary tract cancer.

	Intrahepatic CCA (N = 47)	Perihilar CCA (N = 47)	Distal CCA (N = 24)	Ampulla of Vater CA (N = 26)	Gallbladder CA (N = 61)	Total BTC (N = 205)	*p*-Value
**Stage**							<0.001
I/II	21 (44.7%)	17 (37.8%)	17 (70.8%)	14 (56.0%)	6 (9.8%)	75 (37.1%)	
III/IV	26 (55.3%)	28 (62.2%)	7 (29.2%)	11 (44.0%)	55 (90.2%)	127 (62.9%)	
**Treatment**							<0.001
Surgical Management	12 (25.5%)	10 (21.3%)	10 (43.5%)	20 (76.9%)	29 (47.5%)	81 (39.7%)	
Chemotherapy/Localized Therapy	23 (48.9%)	13 (27.7%)	5 (21.7%)	1 (3.8%)	12 (19.7%)	54 (26.5%)	
Comfort Care	12 (25.5%)	24 (51.1%)	8 (34.8%)	5 (19.2%)	20 (32.8%)	69 (33.8%)	
**Survival**							0.036
Median Survival (Months)	9.0	6.9	10.4	21.4	4.9	7.7	
Survival at 1 Year	42.6%	29.8%	45.8%	69.2%	26.2%	38.5%	
Survival at 5 Years	16.2%	4.0%	5.2%	30.4%	12.1%	12.5%	

**Table 4:** Stage, treatment, and survival by era.

	1976–1990 (N = 46)	1991–2000 (N = 29)	2001–2010 (N = 57)	2011–2018 (N = 73)	1976–2018 (N = 205)	*p*-Value
**Stage**						0.623
I/II	17 (37.8%)	12 (42.9%)	23 (41.1%)	23 (31.5%)	75 (37.1%)	
III/IV	28 (62.2%)	16 (57.1%)	33 (58.9%)	50 (68.5%)	127 (62.9%)	
**Treatment**						<0.001
Surgical Management	23 (51.1%)	13 (44.8%)	26 (45.6%)	19 (26.0%)	81 (39.7%)	
Chemotherapy/Localized Therapy	6 (13.3%)	4 (13.8%)	9 (15.8%)	35 (47.9%)	54 (26.5%)	
Comfort Care	16 (35.6%)	12 (41.4%)	22 (38.6%)	19 (26.0%)	69 (33.8%)	
**Survival**						0.019
Median Survival (Months)	4.2	7.6	7.7	10.8	7.7	
Survival at 1 Year	28.3%	37.9%	40.4%	43.8%	38.5%	
Survival at 5 Years	2.2%	20.7%	11.9%	19.8%	12.5%	

## Data Availability

All data generated or analyzed during this study are included in this article or its Supplementary Materials files. Further inquiries can be directed to the corresponding author.

## Supplementary Materials

The following supporting information can be downloaded at: https://www.mdpi.com/article/10.3390/cancers16152720/s1, Figure S1: age-adjusted incidence rates for biliary tract cancer subtypes; Figure S2: biliary tract cancer survival by subtypes and era; Table S1: Biliary tract cancer codes used in this study. HICDA, Hospital International Classification of Disease Adaptation; ICD, International Classification of Disease; Table S2A–E: patient characteristics, diagnostic, and treatment information categorized into four decennial periods from 1976 to 2018; Table S3: key supplementary statistical analysis for survival outcomes in Figure S2.
