# Supplementary material for: Incidence and Long-Term Outcomes of Biliary Tract Cancers in Olmsted County, Minnesota from 1976 to 2018
Source: Cancers (Basel). 2024 Jul 31;16(15):2720. doi: 10.3390/cancers16152720 (PMC11311608; doi:10.3390/cancers16152720)
Supplement: Supplementary file 1 [file cancers-16-02720-s001.zip › cancers-3052912-supplementary.pdf]

Supplemental Table S1: Biliary tract cancer codes used in the study. HICDA, Hospital International Classification of Disease Adaptation; ICD, International Classification of Disease.

**HICDA code**	**ICD-9 Code**	**ICD-10 Code**
Cholangiocarcinoma (intrahepatic bile duct) (1551211)	Malignant Neoplasm of Intrahepatic Bile Ducts (155.1)	Intrahepatic Bile Duct Carcinoma (C 22.1)
Tumor, Klatskin (1551220)	Malignant Neoplasm of Extrahepatic Bile Ducts (156.1)	Malignant Neoplasm of Gallbladder (C 23)
Neoplasm, Malignant, Hepatic Bile Duct, Primary (1561410)	Malignant Neoplasm of Ampulla of Vater (156.2)	Malignant Neoplasm of Extrahepatic Bile Duct (C 24.0)
Neoplasm, Malignant, Common Bile Duct, Primary (1561210)	Malignant Neoplasm of Gall Bladder (156.0)	Malignant Neoplasm of Ampulla of Vater (C 24.1)
Neoplasm, Malignant, Ampulla of Vater, Primary (1562111)	Malignant Neoplasm of Other Specified Sites of Gall Bladder and Extrahepatic Bile Ducts (156.8)	Malignant Neoplasm of Overlapping Sites of Biliary Tract (C 24.8)
Neoplasm, Malignant, Biliary, NOS, Primary (1569110)	Malignant Neoplasm of Biliary Tract Part Unspecified Site (156.9)	Malignant Neoplasm of Biliary Tract, Unspecified (C 24.9)
Neoplasm, Malignant, Gall Bladder, Primary (1560110)		
History of, Malignancy, Bile Duct (34088-120)		
History of, Malignancy, Ampulla of Vater (34088-121)		
History of, Malignancy, Gall bladder (34088-140)		

Supplemental Table S2A: Patient characteristics, diagnostic, and treatment information for intrahepatic CCA categorized into four decennial periods from 1976 to 2018.

	1976-1900 (N=2)	1991-2000 (N=4)	2001-2010 (N=13)	2011-2018 (N=28)	Total (N=47)	P-value
**Demographics**						
Median Age (Range)	65.9 (62.9, 69.0)	59.6 (42.6, 84.9)	68.0 (41.2, 92.8)	65.6 (46.2, 96.5)	66.5 (41.2, 96.5)	0.7073[1]
Age of Diagnosis < 50	0 (0.0%)	1 (7.7%)	1 (7.7%)	2 (7.1%)	4 (8.5%)	0.6456[2]
Female, N (%)	1 (50.0%)	2 (50.0%)	3 (23.1%)	11 (39.3%)	17 (36.2%)	0.6639[2]
White, N (%)	2 (100.0%)	4 (100.0%)	12 (92.3%)	25 (89.3%)	43 (91.5%)	0.8628[2]
**Stage**, n(%)						0.6353[2]
Stage 1-2	0 (0.0%)	2 (50.0%)	6 (46.2%)	13 (46.4%)	21 (44.7%)	
Stage 3-4	2 (100.0%)	2 (50.0%)	7 (53.8%)	15 (53.6%)	26 (55.3%)	
Missing						
**Diagnosis Method**						0.0026[2]
Histology, N (%)	2 (100.0%)	3 (75.0%)	5 (38.5%)	27 (96.4%)	37 (78.7%)	
Cytology, N (%)	0 (0.0%)	1 (25.0%)	7 (53.8%)	0 (0.0%)	8 (17.0%)	
Radiology, N (%)	0 (0.0%)	0 (0.0%)	1 (7.7%)	1 (3.6%)	2 (4.3%)	
**Risk Factors**						
Porcelain Gallbladder, N (%)	-	-	-	-	-	
Gallstones, N (%)	-	-	-	-	-	
Primary Sclerosing Cholangitis, N (%)	0 (0.0%)	0 (0.0%)	1 (7.7%)	1 (3.6%)	2 (4.3%)	0.8789[2]
Cirrhosis, N (%)	0 (0.0%)	1 (25.0%)	4 (30.8%)	6 (21.4%)	11 (23.4%)	0.7841[2]
Alcohol Use, N (%)	1 (50.0%)	0 (0.0%)	1 (7.7%)	4 (14.3%)	6 (12.8%)	0.3295[2]
Smoking, N (%)*	2 (100.0%)	2 (50.0%)	9 (75.0%)	13 (46.4%)	26 (56.5%)	0.2181[2]
Missing						
Family History, N (%)*	-	-	-	-	-	
Missing						
HBV, N (%)	0 (0.0%)	0 (0.0%)	1 (7.7%)	1 (3.6%)	2 (4.3%)	0.8789[2]
HCV, N (%)	0 (0.0%)	0 (0.0%)	0 (0.0%)	1 (3.6%)	1 (2.1%)	0.8748[2]
IBD	0 (0.0%)	0 (0.0%)	1 (7.7%)	0 (0.0%)	1 (2.1%)	0.4450[2]
**Initial Treatment**						0.9318[2]
Surgery	1 (50.0%)	1 (25.0%)	4 (30.8%)	6 (21.4%)	12 (25.5%)	

Chemotherapy/Radiation	1 (50.0%)	2 (50.0%)	5 (38.5%)	15 (53.6%)	23 (48.9%)	
Pallative	0 (0.0%)	1 (25.0%)	4 (30.8%)	7 (25.0%)	12 (25.5%)	
Missing Data						
**Detailed Treatment Information**						0.7008[2]
Curative resection	0 (0.0%)	1 (25.0%)	1 (10.0%)	0 (%)	2 (12.5%)	
Liver transplant	0 (0.0%)	0 (0.0%)	1 (10.0%)	0 (%)	1 (6.3%)	
Non cuarative resection	1 (50.0%)	0 (0.0%)	1 (10.0%)	0 (%)	2 (12.5%)	
Systematic Chemotherapy	1 (50.0%)	2 (50.0%)	3 (30.0%)	0 (%)	6 (37.5%)	
Comfort	0 (0.0%)	1 (25.0%)	4 (40.0%)	0 (%)	5 (31.3%)	
**BTC Related Death**						0.8595[2]
1.BTC Related Death	2 (100.0%)	3 (100.0%)	12 (100.0%)	22 (95.7%)	39 (97.5%)	
Missing	0	1	1	5	7	
[1]Kruskal-Wallis p-value; [2]Chi-Square p-value;						

Supplemental Table S2B. Patient characteristics, diagnostic, and treatment information for perihilar CCA categorized into four decennial periods from 1976 to 2018.

	Perihilar CCA					
	1976-1990 (N=12)	1991-2000 (N=9)	2001-2010 (N=11)	2011-2018 (N=15)	Total (N=47)	**P-value**
**Demographics**						
Median Age (Range)	66.3 (52.1, 96.3)	70.9 (37.0, 85.0)	69.8 (47.2, 87.9)	81.3 (49.9, 91.9)	70.9 (37.0, 96.3)	0.2642[1]
Age of Diagnosis < 50	0 (0.0%)	1 (11.1%)	1 (9.1%)	1 (6.7%)	3 (6.4%)	0.7311[2]
Female, N (%)	6 (50.0%)	3 (33.3%)	7 (63.6%)	7 (46.7%)	23 (48.9%)	
White, N (%)	12 (100.0%)	9 (100.0%)	11 (100.0%)	15 (100.0%)	47 (100.0%)	
**Stage**, n(%)						0.0586[2]
Stage 1-2	7 (63.6%)	4 (50.0%)	4 (36.4%)	2 (13.3%)	17 (37.8%)	
Stage 3-4	4 (36.4%)	4 (50.0%)	7 (63.6%)	13 (86.7%)	28 (62.2%)	
Missing	1	1	0	0	2	
**Diagnosis Method**						0.1768[2]
Histology, N (%)	11 (91.7%)	4 (44.4%)	5 (45.5%)	8 (53.3%)	28 (59.6%)	
Cytology, N (%)	0 (0.0%)	3 (33.3%)	4 (36.4%)	6 (40.0%)	13 (27.7%)	
Radiology, N (%)	1 (8.3%)	2 (22.2%)	2 (18.2%)	1 (6.7%)	6 (12.8%)	
**Risk Factors**						
Porcelain Gallbladder, N (%)	-	-	-	-	-	
Gallstones, N (%)	5 (41.7%)	5 (55.6%)	2 (18.2%)	10 (66.7%)	22 (46.8%)	0.0937[2]
Primary Sclerosing Cholangitis, N (%)	0 (0.0%)	0 (0.0%)	3 (27.3%)	3 (20.0%)	6 (12.8%)	0.1188[2]
Cirrhosis, N (%)	2 (16.7%)	1 (11.1%)	1 (9.1%)	1 (6.7%)	5 (10.6%)	0.8644[2]
Alcohol Use, N (%)	1 (8.3%)	2 (22.2%)	0 (0.0%)	1 (6.7%)	4 (8.5%)	0.3529[2]
Smoking, N (%)*	9 (75.0%)	6 (66.7%)	7 (63.6%)	9 (60.0%)	31 (66.0%)	0.8726[2]
Missing						
Family History, N (%)*	-	-	-	-	-	
Missing						
HBV, N (%)	-	-	-	-	-	

HCV, N (%)	-	-	-	-	-	
IBD	1 (8.3%)	0 (0.0%)	3 (27.3%)	3 (20.0%)	7 (14.9%)	0.3054[2]
**Initial Treatment**						0.1501[2]
Surgery	4 (33.3%)	1 (11.1%)	3 (27.3%)	2 (13.3%)	10 (21.3%)	
Chemotherapy/Radiation	2 (16.7%)	1 (11.1%)	2 (18.2%)	8 (53.3%)	13 (27.7%)	
Pallative	6 (50.0%)	7 (77.8%)	6 (54.5%)	5 (33.3%)	24 (51.1%)	
Missing Data						
**Detailed Treatment Information**						0.3344[2]
Curative resection	3 (25.0%)	1 (11.1%)	1 (14.3%)	0 (%)	5 (17.9%)	
Liver transplant	0 (0.0%)	0 (0.0%)	2 (28.6%)	0 (%)	2 (7.1%)	
Non cuarative resection	1 (8.3%)	0 (0.0%)	0 (0.0%)	0 (%)	1 (3.6%)	
Systematic Chemotherapy	2 (16.7%)	1 (11.1%)	1 (14.3%)	0 (%)	4 (14.3%)	
Comfort	6 (50.0%)	7 (77.8%)	3 (42.9%)	0 (%)	16 (57.1%)	
**BTC Related Death**						
1.BTC Related Death	12 (100.0%)	9 (100.0%)	9 (100.0%)	13 (100.0%)	43 (100.0%)	
Missing	0	0	2	2	4	
[1]Kruskal-Wallis p-value; [2]Chi-Square p-value;						

Supplemental Table S2C. Patient characteristics, diagnostic, and treatment information for Distal Extrahepatic CCA categorized into four decennial periods from 1976 to 2018.

	1976-1990 (N=6)	1991-2000 (N=3)	2001-2010 (N=8)	2011-2018 (N=7)	Total (N=24)	**P-value**
Demographics						
**Median Age (Range)**	71.6 (51.4, 87.1)	63.5 (56.1, 64.6)	78.2 (69.3, 94.8)	66.1 (41.7, 87.8)	70.2 (41.7, 94.8)	0.0744[1]
Age of Diagnosis < 50	0 (0.0%)	0 (0.0%)	0 (0.0%)	1 (14.3%)	1 (4.2%)	0.4691[2]
Female, N (%)	2 (33.3%)	3 (100.0%)	2 (25.0%)	4 (57.1%)	11 (45.8%)	0.1281[2]
White, N (%)	6 (100.0%)	2 (66.7%)	8 (100.0%)	7 (100.0%)	23 (95.8%)	0.0628[2]
**Stage**, n(%)						0.7575[2]
Stage 1-2	5 (83.3%)	2 (66.7%)	6 (75.0%)	4 (57.1%)	17 (70.8%)	
Stage 3-4	1 (16.7%)	1 (33.3%)	2 (25.0%)	3 (42.9%)	7 (29.2%)	
Missing						
Diagnosis Method						0.1884[2]
Histology, N (%)	4 (66.7%)	3 (100.0%)	6 (75.0%)	5 (71.4%)	18 (75.0%)	
Cytology, N (%)	0 (0.0%)	0 (0.0%)	2 (25.0%)	2 (28.6%)	4 (16.7%)	
**Radiology, N (%)**	2 (33.3%)	0 (0.0%)	0 (0.0%)	0 (0.0%)	2 (8.3%)	
**Risk Factors**						
Porcelain Gallbladder, N (%)	-	-	-	-		
Gallstones, N (%)	2 (33.3%)	0 (0.0%)	4 (50.0%)	4 (57.1%)	10 (41.7%)	0.3571[2]
**Primary Sclerosing Cholangitis, N (%)**	-	-	-	-	-	
Cirrhosis, N (%)	-	-	-	-	-	

Alcohol Use, N (%)	0 (0.0%)	0 (0.0%)	1 (12.5%)	2 (28.6%)	3 (12.5%)	0.4012[2]
Smoking, N (%)*	5 (83.3%)	2 (66.7%)	5 (71.4%)	4 (57.1%)	16 (69.6%)	0.7841[2]
Missing	0	0	1	0	1	
Family History, N (%)*	-	-	-	-	-	
Missing						
HBV, N (%)	-	-	-	-	-	
HCV, N (%)	-	-	-	-	-	
IBD	-	-	-	-	-	
**Initial Treatment**						0.7617[2]
Surgery	2 (40.0%)	2 (66.7%)	4 (50.0%)	2 (28.6%)	10 (43.5%)	
Chemotherapy/Radiation	1 (20.0%)	0 (0.0%)	1 (12.5%)	3 (42.9%)	5 (21.7%)	
Palliative	2 (40.0%)	1 (33.3%)	3 (37.5%)	2 (28.6%)	8 (34.8%)	
Missing Data	1	0	0	0	0	1
**Detailed Treatment Information**						0.7383[2]
Curative resection	0 (0.0%)	1 (33.3%)	3 (42.9%)	0 (%)	4 (26.7%)	
Liver transplant	-	-	-	-	-	
Non curative resection	2 (40.0%)	1 (33.3%)	1 (14.3%)	0 (%)	4 (26.7%)	
Systematic Chemotherapy	1 (20.0%)	0 (0.0%)	1 (14.3%)	0 (%)	2 (13.3%)	
Comfort	2 (40.0%)	1 (33.3%)	2 (28.6%)	0 (%)	5 (33.3%)	
BTC Related Death						0.5807[2]
1.BTC Related Death	6 (100.0%)	3 (100.0%)	7 (87.5%)	6 (100.0%)	22 (95.7%)	
Missing	0	0	0	0	1	1

[1]Kruskal-Wallis p-value; [2]Chi-Square p-value;

Supplemental Table S2D. Patient characteristics, diagnostic, and treatment information for Ampullary Cancer categorized into four decennial periods from 1976 to 2018.

Ampullary CCA						
	**1976-1990(N =4)**	**1991-2000(N=3)**	**2001-2010(N=12)**	**2011-2018(N=7)**	**Total(N=26)**	**P-value**
**Demographics**						
Median Age (Range)	74.5 (69.7, 89.7)	70.0 (56.8, 80.0)	70.4 (45.2, 89.4)	73.6 (38.5, 85.4)	70.4 (38.5, 89.7)	0.8223[1]
Age of Diagnosis < 50	0 (0.0%)	0 (0.0%)	1 (8.3%)	1 (14.3%)	2 (7.7%)	
Female, N (%)	2 (50.0%)	2 (66.7%)	3 (25.0%)	4 (57.1%)	11 (42.3%)	0.4025[2]
White, N (%)	4 (100.0%)	3 (100.0%)	12 (100.0%)	7 (100.0%)	26 (100.0%)	
**Stage, n(%)**						0.1430[2]
Stage 1-2	4 (100.0%)	2 (66.7%)	6 (54.5%)	2 (28.6%)	14 (56.0%)	
Stage 3-4	0 (0.0%)	1 (33.3%)	5 (45.5%)	5 (71.4%)	11 (44.0%)	
Missing						
Diagnosis Method						0.7498[2]
Histology, N (%)	0 (0.0%)	0 (0.0%)	1 (8.3%)	0 (0.0%)	1 (3.8%)	
Cytology, N (%)	4 (100.0%)	3 (100.0%)	11 (91.7%)	7 (100.0%)	25 (96.2%)	
Radiology, N (%)	-	-	-	-	-	
**Risk Factors**						
Porcelain Gallbladder, N (%)	-	-	-	-	-	
Gallstones, N (%)	1 (25.0%)	2 (66.7%)	5 (41.7%)	4 (57.1%)	12 (46.2%)	0.6446[2]
Primary Sclerosing Cholangitis, N (%)	-	-	-	-	-	

Cirrhosis, N (%)	-	-	-	-	-	
Alcohol Use, N (%)	0 (0.0%)	0 (0.0%)	1 (8.3%)	1 (14.3%)	2 (7.7%)	0.7967[2]
Smoking, N (%)*	2 (50.0%)	2 (66.7%)	7 (58.3%)	4 (57.1%)	15 (57.7%)	0.9778[2]
Family History, N (%)*						
HBV, N (%)	-	-	-	-	-	
HCV, N (%)	-	-	-	-	-	
IBD	-	-	-	-	-	
**Initial Treatment**						0.3808[2]
Surgery	3 (75.0%)	3 (100.0%)	8 (66.7%)	6 (85.7%)	20 (76.9%)	
Chemotherapy/Radiation	0 (0.0%)	0 (0.0%)	0 (0.0%)	1 (14.3%)	1 (3.8%)	
Pallative	1 (25.0%)	0 (0.0%)	4 (33.3%)	0 (0.0%)	5 (19.2%)	
**Detailed Treatment Information**						
Curative resection	3 (75.0%)	1 (33.3%)	2 (28.6%)	0 (%)	6 (42.9%)	
Liver transplant	-	-	-	-	-	
Non cuarative resection	0 (0.0%)	2 (66.7%)	2 (28.6%)	0 (%)	4 (28.6%)	
Systematic Chemotherapy						
Comfort	1 (25.0%)	0 (0.0%)	3 (42.9%)	0 (%)	4 (28.6%)	
**BTC Related Death**						0.4240[2]
BTC Related Death	2 (50.0%)	2 (100.0%)	7 (77.8%)	2 (100.0%)	13 (76.5%)	
Missing	0	1	3	5	9	
[1]Kruskal-Wallis p-value; [2]Chi-Square p-value;						

Supplemental Table S2E. Patient characteristics, diagnostic, and treatment information for Gallbladder Cancer categorized into four decennial periods from 1976 to 2018.

	1976-1990(N=22)	1991-2000(N=10)	2001-2010(N=13)	2011-2018(N=16)	Total(N=61)	P-value
**Demographics**						
Median Age (Range)	77.1 (47.5, 89.8)	72.4 (57.5, 87.7)	69.2 (44.6, 85.6)	77.0 (48.8, 87.6)	72.8 (44.6, 89.8)	0.4376[1]
Age of Diagnosis < 50	2 (9.1%)	0 (0.0%)	2 (15.4%)	1 (6.3%)	5 (8.2%)	0.5957[2]
Female, N (%)	17 (77.3%)	8 (80.0%)	7 (53.8%)	6 (37.5%)	38 (62.3%)	0.0456[2]
White, N (%)	22 (100.0%)	10 (100.0%)	12 (92.3%)	15 (93.8%)	59 (96.7%)	0.5070[2]
**Stage, n(%)**						0.5612[2]
Stage 1-2	1 (4.5%)	2 (20.0%)	1 (7.7%)	2 (12.5%)	6 (9.8%)	
Stage 3-4	21 (95.5%)	8 (80.0%)	12 (92.3%)	14 (87.5%)	55 (90.2%)	
**Diagnosis Method**						
Histology, N (%)						0.03872
Cytology, N (%)	20 (90.9%)	7 (70.0%)	8 (61.5%)	10 (62.5%)	45 (73.8%)	
Radiology, N (%)	0 (0.0%)	3 (30.0%)	5 (38.5%)	3 (18.8%)	11 (18.0%)	
	2 (9.1%)	0 (0.0%)	0 (0.0%)	3 (18.8%)	5 (8.2%)	
**Risk Factors**						
Porcelain Gallbladder, N (%)						
Gallstones, N (%)						
Primary Sclerosing Cholangitis, N (%)	19 (86.4%)	7 (70.0%)	11 (84.6%)	8 (50.0%)	45 (73.8%)	0.0618[2]

Cirrhosis, N (%)	0 (0.0%)	0 (0.0%)	0 (0.0%)	1 (6.3%)	1 (1.6%)	0.4138[2]
Alcohol Use, N (%)						
Smoking, N (%)*						0.1094[2]
Missing	8 (36.4%)	7 (77.8%)	8 (61.5%)	6 (37.5%)	29 (48.3%)	*
Family History, N (%)*	0	1	0	0	1	
Missing	1 (4.8%)	0 (0.0%)	1 (7.7%)	0 (0.0%)	2 (3.3%)	0.6158[2]
HBV, N (%)	1	0	0	0	1	
HCV, N (%)	-	-	-	-	-	
IBD	-	-	-	-	-	
	0 (0.0%)	1 (10.0%)	0 (0.0%)	3 (18.8%)	4 (6.6%)	0.0884[2]
**Initial Treatment**						
Surgery						0.0278[2]
Chemotherapy/Radiation	13 (59.1%)	6 (60.0%)	7 (53.8%)	3 (18.8%)	29 (47.5%)	
Pallative	2 (9.1%)	1 (10.0%)	1 (7.7%)	8 (50.0%)	12 (19.7%)	
Missing Data	7 (31.8%)	3 (30.0%)	5 (38.5%)	5 (31.3%)	20 (32.8%)	
**Detailed Treatment Information**						
Curative resection						0.9287[2]
Liver transplant	5 (22.7%)	4 (40.0%)	3 (30.0%)	0 (%)	12 (28.6%)	
Non cuarative resection	-	-	-	-	-	
Systematic Chemotherapy	8 (36.4%)	2 (20.0%)	2 (20.0%)	0 (%)	12 (28.6%)	
Comfort	2 (9.1%)	1 (10.0%)	1 (10.0%)	0 (%)	4 (9.5%)	
**BTC Related Death**	7 (31.8%)	3 (30.0%)	4 (40.0%)	0 (%)	14 (33.3%)	
BTC Related Death						0.7488[2]

Missing	19 (90.5%)	8 (88.9%)	11 (100.0%)	13 (92.9%)	51 (92.7%)	
1Kruskal-Wallis p-value; 2Chi-Square p-value;	1	1	2	2	6	

Supplemental Table S3: Key supplementary statistical analysis for survival outcomes in Figure S2

Subgroup	Era	N (Events)	Median Survival (95% CI)	HR (95% CI) Compared to 1976-1990	Log rank P-value
All Patients	1976-1990	46 (45)	4.22 (1.91, 7.65)	Reference	p = 0.035
	1991-2000	29 (26)	7.56 (5.75, 32.26)	0.54 (0.32 - 0.89)	
	2001-2010	57 (49)	7.65 (4.96, 17.54)	0.63 (0.42 - 0.95)	
	2011-2018	73 (58)	10.84 (9.00, 16.16)	0.57 (0.39 - 0.85)	
Intrahepatic CCA	1976-1990	2 (2)	9.86 (1.91, NE)	Reference	p = 0.8
	1991-2000	4 (3)	4.70 (1.77, NE)	0.54 (0.32 - 0.89)	
	2001-2010	13 (12)	4.76 (1.18, NE)	0.63 (0.42 - 0.95)	
	2011-2018	28 (23)	10.69 (4.86, 22.96)	0.57 (0.39 - 0.85)	
Perihiliar CCA	1976-1990	12 (12)	4.27 (1.68, NE)	Reference	p = 0.8
	1991-2000	9 (9)	6.90 (3.88, NE)	0.54 (0.32 - 0.89)	
	2001-2010	11 (9)	7.65 (5.72, NE)	0.63 (0.42 - 0.95)	
	2011-2018	15 (13)	6.57 (2.56, NE)	0.57 (0.39 - 0.85)	
Gallbladder CCA	1976-1990	22 (21)	2.89 (1.38, 6.57)	Reference	p = 0.278
	1991-2000	10 (9)	6.39 (2.37, NE)	0.54 (0.32 - 0.89)	
	2001-2010	13 (11)	5.88 (1.64, NE)	0.63 (0.42 - 0.95)	
	2011-2018	16 (14)	10.10 (3.02, NE)	0.57 (0.39 - 0.85)	
Distal CCA	1976-1990	6 (6)	11.15 (0.49, NE)	Reference	p = 0.185
	1991-2000	3 (3)	55.72 (9.89, NE)	0.54 (0.32 - 0.89)	
	2001-2010	8 (8)	6.96 (1.61, NE)	0.63 (0.42 - 0.95)	
	2011-2018	7 (6)	12.48 (3.29, NE)	0.57 (0.39 - 0.85)	
Ampullary CCA	1976-1990	4 (4)	12.24 (0.20, NE)	Reference	p = 0.121
	1991-2000	3 (2)	12.91 (9.36, NE)	0.54 (0.32 - 0.89)	

	2001-2010	12 (9)	18.79 (5.09, NE)	0.63 (0.42 - 0.95)	
	2011-2018	7 (2)	NE (21.42, NE)	0.57 (0.39 - 0.85)	

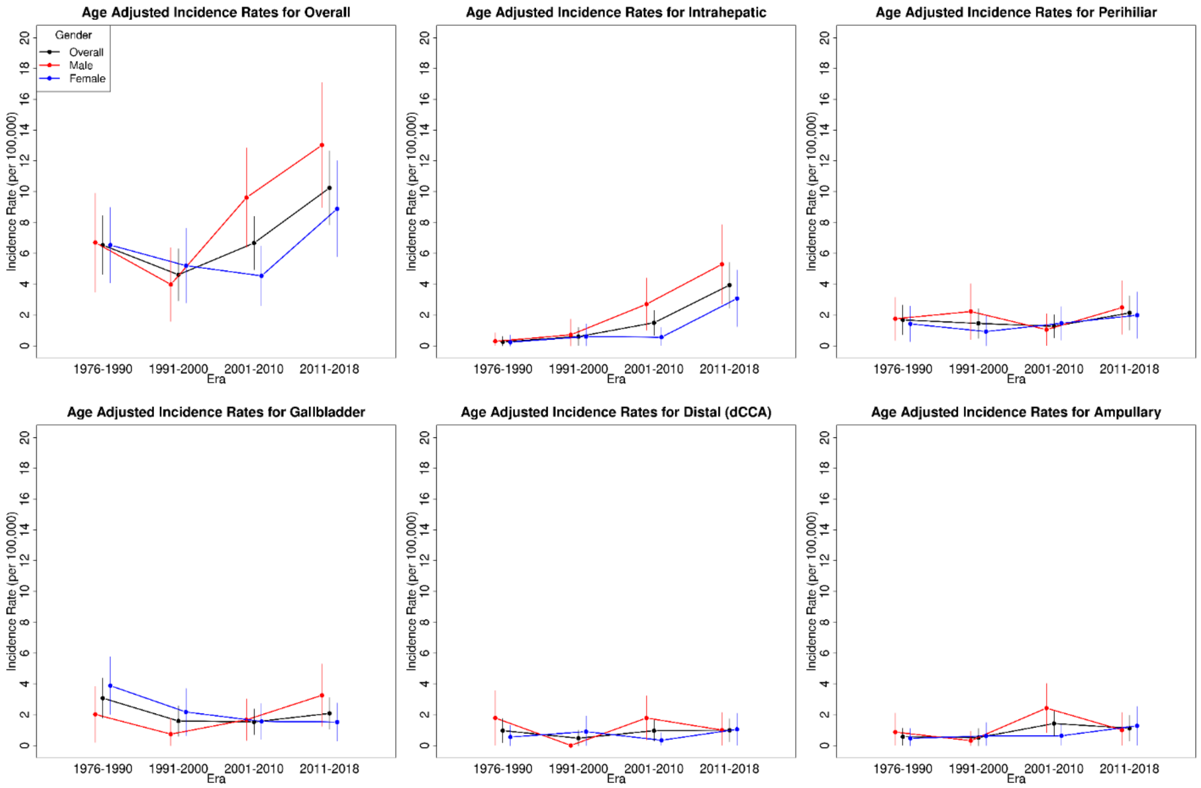

Supplemental Figure S1: Age-adjusted incidence rates for biliary tract cancer subtypes.

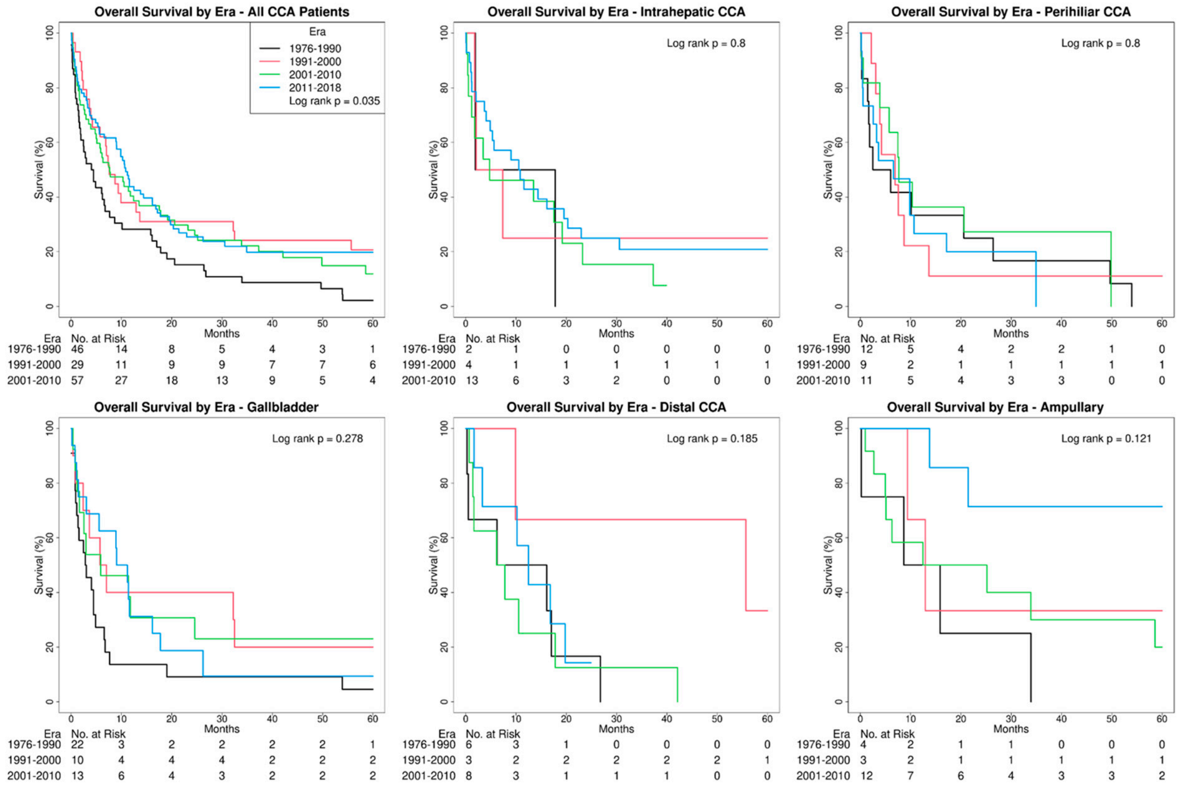

Supplemental Figure S2: Survival by Era for each subtype.
